# Diet and Lp(a): Does Dietary Change Modify Residual Cardiovascular Risk Conferred by Lp(a)?

**DOI:** 10.3390/nu12072024

**Published:** 2020-07-07

**Authors:** Byambaa Enkhmaa, Kristina S. Petersen, Penny M. Kris-Etherton, Lars Berglund

**Affiliations:** 1 Department of Internal Medicine, School of Medicine, University of California Davis, Davis, CA 95616, USA; ebyambaa@ucdavis.edu; 2 Department of Nutritional Sciences, The Pennsylvania State University, University Park, PA 16802, USA; kup63@psu.edu (K.S.P.); pmk3@psu.edu (P.M.K.-E.)

**Keywords:** diet, Lp(a), cardiovascular risk, metabolic feeding trials

## Abstract

Lipoprotein(a) [Lp(a)] is an independent, causal, genetically determined risk factor for cardiovascular disease (CVD). We provide an overview of current knowledge on Lp(a) and CVD risk, and the effect of pharmacological agents on Lp(a). Since evidence is accumulating that diet modulates Lp(a), the focus of this paper is on the effect of dietary intervention on Lp(a). We identified seven trials with 15 comparisons of the effect of saturated fat (SFA) replacement on Lp(a). While replacement of SFA with carbohydrate, monounsaturated fat (MUFA), or polyunsaturated fat (PUFA) consistently lowered low-density lipoprotein cholesterol (LDL-C), heterogeneity in the Lp(a) response was observed. In two trials, Lp(a) increased with carbohydrate replacement; one trial showed no effect and another showed Lp(a) lowering. MUFA replacement increased Lp(a) in three trials; three trials showed no effect and one showed lowering. PUFA or PUFA + MUFA inconsistently affected Lp(a) in four trials. Seven trials of diets with differing macronutrient compositions showed similar divergence in the effect on LDL-C and Lp(a). The identified clinical trials show diet modestly affects Lp(a) and often in the opposing direction to LDL-C. Further research is needed to understand how diet affects Lp(a) and its properties, and the lack of concordance between diet-induced LDL-C and Lp(a) changes.

## 1. Introduction

Cardiovascular disease (CVD) is the number one cause of death and disability worldwide accounting for 32% of all deaths and 15% of disability adjusted life-years [1]. Notably, poor diet quality accounts for a substantial proportion of this cardiovascular related death and disability. Worldwide and in the U.S., poor diet quality is the leading cause of death [1,2]. Globally, 22% of all deaths are diet-related, although 53% of CVD deaths are attributed to dietary risks [3]. Similarly, in the U.S., 18% of deaths are attributed to dietary risks, with 48% of CVD deaths caused by poor diet [3]. Poor diet quality is associated with overweight and obesity, dyslipidemia, hypertension and dysglycemia [4]. While the effect of diet on total cholesterol, low-density lipoprotein cholesterol (LDL-C), triglycerides, and high-density lipoprotein cholesterol (HDL-C) is relatively well-characterized [5,6,7], less is known about dietary modulation of lipoprotein(a) [Lp(a)].

The overarching aim of this review is to summarize clinical trial evidence examining the effect of dietary change on Lp(a), and to compare this with the effect of dietary interventions on LDL-C, an established target for CVD prevention and management [8]. Firstly, for context, we will provide an overview of the current state of knowledge on the role of Lp(a) in the development of CVD, particularly related to residual cardiovascular risk. In addition, we will briefly describe the latest evidence for the efficacy of established lipid-lowering therapies to reduce Lp(a). Thus, this review will provide an up-to-date summary of current evidence on Lp(a) and pharmacological and non-pharmacological therapies being tested for Lp(a) management to reduce CVD risk.

## 2. Lipoprotein(a) Structure and Unique Features

**Figure 1:**
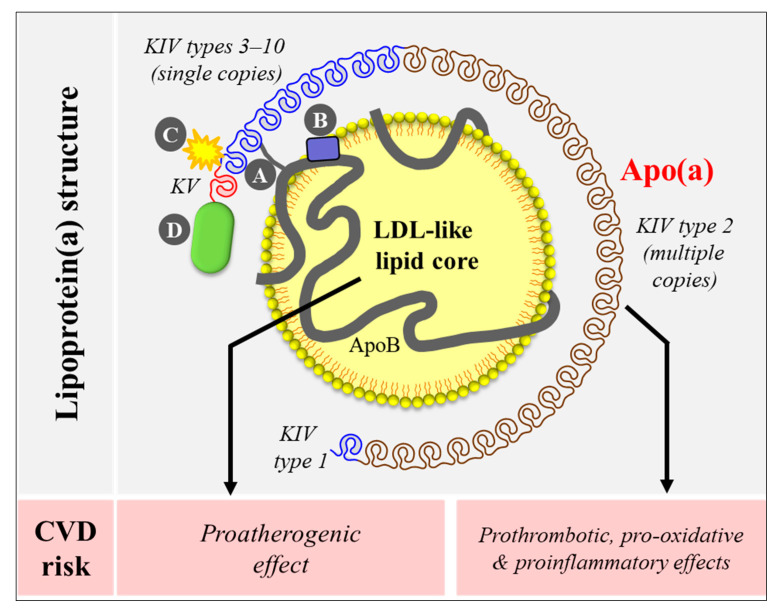
Lipoprotein(a) [Lp(a)] structure and potential mechanisms underlying cardiovascular risk. Lp(a) contains an LDL-like core and one molecule of apolipoprotein(a) (apo(a)). Apo(a) binds to apoB-100 of the LDL-like core via a single disulfide bond (**A**) at a location near the LDL receptor binding site (**B**). Apo(a) has repeated kringle (K) structures (KIV and KV) similar to that of the plasminogen gene. Apo(a) KIV has 10 different types, of which type 2 is present in multiple copies. Apo(a) binds to proinflammatory and proatherogenic oxidized phospholipids via its KIV type 10 (**C**) [16,17]. Apo(a) also has a protease domain (**D**) that lacks proteolytic activity. Lp(a) promotes cardiovascular risk through proatherogenic (via its LDL-like core) and prothrombotic/ proinflammatory (via its apo(a)) mechanisms.

Research spanning over several decades has established the importance of Lp(a) in human health and disease. While the exact physiological function of Lp(a) remains to be determined, the pathophysiological role of Lp(a) as an independent causal risk factor CVD has been well established. Lp(a) was first detected in 1963 by Berg as an antigen-like material when rabbits were immunized with a human low-density lipoprotein (LDL) sample containing high Lp(a) [9]. Like an LDL particle, Lp(a) has a cholesteryl-ester rich lipid core and one molecule of apolipoprotein B-100 (apoB). This unit forms a covalent bridge with another hepatically produced apolipoprotein called apo(a) that gives many unique features to Lp(a). Apo(a) contains coding sequences forming multiple tri-loop structures termed Kringles (K) structurally similar to that of the plasminogen gene [10]. Although plasminogen contains five K domains, only two of them (KIV/KV) are present in the human *LPA* gene, where KIV is differentiated into 10 subtypes (KIV type 1–10). Of these, KIV type 2 motif is repeated multiple times (3 to > 40 copies), resulting in an extensive size heterogeneity in the apo(a) gene and consequently in the apo(a) protein [11,12,13,14,15]. Lp(a) structure is shown in Figure 1 (upper panel). Lp(a) is thought to promote CVD risk through proatherogenic (via its LDL-like lipid core) and prothrombotic/proinflammatory (via its apo(a)) pathways (Figure 1, lower panel).

The apo(a) size polymorphism is considered to be a major genetic regulator of plasma Lp(a) concentration as a strong inverse association exists between the number of KIV type 2 copies (i.e., the size) and the circulating level of Lp(a) [18,19]. Interestingly, although the distributions of apo(a) sizes at the DNA level (alleles) and at the posttranslational level (isoforms) do not differ substantially between population groups, a consistent interethnic/ interracial difference has been observed for plasma Lp(a) concentration. Thus, African populations display, on average, a 2- to 3-fold higher plasma Lp(a) concentration compared to non-Africans. This observation suggests a complex ethnicity/race-specific genetic regulation of Lp(a) and a role for other genetic as well as non-genetic factors in modulating Lp(a) concentration.

## 3. Lipoprotein(a) [Lp(a)] Is an Independent, Causal, Genetically Determined Cardiovascular Disease (CVD) Risk Factor

A large body of concordant evidence shows that an elevated level of plasma Lp(a)—a highly-heritable trait—is independently and causally associated with CVD. The findings in early case-control studies linking Lp(a) with coronary heart disease (CHD) have been confirmed in subsequent prospective, genetic epidemiological, and Mendelian randomization studies [20,21,22,23,24,25,26]. Several original and updated meta-analyses based on a large number of prospective studies compiling data from a few thousand to >100 thousand participants have found significant associations between Lp(a) and CHD. The findings of these meta-analyses demonstrate a 70% increased risk of CHD in subjects in the top vs. the bottom tertile of Lp(a) [27], a persistent independent and continuous association of Lp(a) with the risk of future CHD after adjusting for established risk factors [28] and a continuous association of Lp(a) level with the risks of CHD and stroke independent of traditional risk factors [29]. Studies using Mendelian randomization approaches based on the apo(a) size polymorphism have provided support for the causal role of Lp(a) in CHD. The presence of a small apo(a) size genotype was associated with both a high Lp(a) level phenotype and the presence of CHD [30,31,32,33]. Consistent with these findings, with increasing numbers of KIV repeats, Lp(a) levels decreased as expected, and an increase in risk of myocardial infarction (MI) was observed with increasing Lp(a) levels as well as with decreasing numbers of KIV repeats [32]. In another study using the same Mendelian randomization, Lp(a) levels and apo(a) KIV repeat tertiles were associated with risks of coronary, carotid and femoral atherosclerotic stenosis [33], providing mechanistic insights into Lp(a) pathogenicity. Another meta-analysis demonstrated that carriers of small apo(a) isoforms have a 2-fold higher risk of CHD or ischemic stroke compared with carriers of large isoforms [34].

Single nucleotide polymorphisms (SNPs) in the *LPA* (e.g., rs10455872 and rs3798220) and non-*LPA* genes with significant effects on Lp(a) concentrations [26,35,36,37] have been associated with CHD or severe coronary stenosis [38,39,40,41,42,43,44]. The two *LPA* SNPs, rs10455872 and rs3798220, were also associated with aortic valve calcification in a multi-ethnic cohort [45] as well as with peripheral artery disease (PAD), abdominal aortic aneurysm and large artery atherosclerosis subtype of ischemic stroke [46]. The association between Lp(a) and calcified aortic valve disease has been increasingly recognized and the findings in early epidemiological (cross-sectional, case-control and cohort) studies demonstrating an association of Lp(a) with the disease [47,48,49,50] have been further confirmed in subsequent genetic association studies [45,51,52]. The presence of two copies of the rs10455872 G allele was associated with a hazard ratio (HR) of 4.83 for incident calcified aortic valve disease [52]. Further, Lp(a) has been shown to carry the majority of proinflammatory and proatherogenic oxidized phospholipids (OxPL), particularly phosphocholine-containing ones, in the circulation [53] and evidence suggests that this function of Lp(a) mediates its pathogenicity. The level of OxPL carried on apoB-containing lipoproteins predicted CVD risk [54,55] and was associated with faster aortic stenosis aggravation and the need for aortic valve replacement [56]. Taken together, a large body of data forms the evidence base for clinical guidelines for CVD risk reduction to evaluate Lp(a) levels.

## 4. Public Health and Clinical Relevance of Lp(a) as a Contributor to Residual CVD Risk

Since 2016, several national or international guidelines and consensus statements on Lp(a) testing and treatment have been published. These guidelines issued by authorities such as the American College of Cardiology (ACC)/American Heart Association (AHA) Task Force [8], the American Society for Apheresis [57], the Canadian Cardiovascular Society [58], the National Lipid Association [59], and the HEART UK Medical, Scientific and Research Committee [60] are in general agreement to measure Lp(a) in individuals at intermediate to high risk for CVD and those with family history of premature CVD and define Lp(a) risk threshold at > 30 mg/dL to > 50 mg/dL (>75 nmol/L to >125 nmol/L). In addition, the 2019 European Society of Cardiology and European Atherosclerosis Society guideline recommends that Lp(a) levels should be measured at least once in each adult person’s lifetime to identify those with very high inherited Lp(a) levels >180 mg/dL (>430 nmol/L) who may have a lifetime risk of CVD comparable to those with heterozygous familial hypercholesterolemia (FH) [61]. The ACC/AHA Task Force on Clinical Practice Guideline recognizes Lp(a) as a risk-enhancing factor at levels >50 mg/dL (>125 nmol/L) [8]. Regarding therapeutic guidance, the American Society for Apheresis consensus recommends nicotinic acid (1–3 g/day) as the first-line of treatment, and if refractory, weekly selective lipid apheresis to lower Lp(a) [57].

Moving beyond the role of Lp(a) in CVD risk in the general population, a recent meta-analysis attempted to clarify Lp(a)-attributable residual CVD risk in patients with established CVD or on statin therapy [62]. This study using patient-level data from seven placebo-controlled statin trials encompassing 29,069 patients analyzed the relation of baseline and on-treatment Lp(a) concentration to risk of major adverse cardiovascular events (MACE). Statin therapy, as expected, reduced LDL-C level; after accounting for the contribution of Lp(a) the degree of reduction was 39%. However, the statin effect on Lp(a) was heterogeneous with three trials showing an increase (2% to 15%) and four trials showing a decrease (−1% to −13%) [62]. Elevated Lp(a) concentration exceeding 50 mg/dL at baseline or on-treatment was associated with an increased HR of MACE independent of other CVD risk factors. Interestingly, this association was stronger in patients receiving statins than those on placebo, suggesting that residual risk is present in patients with elevated Lp(a) that is not addressed by statins [62]. In patients with elevated Lp(a) levels who managed their LDL-C-attributable risk with statin therapy, specific therapies to lower Lp(a) may alleviate Lp(a)-induced CVD risk.

## 5. Lp(a), Lipid-Lowering Therapeutics and Cardiovascular Benefit

Apart from lipid apheresis that induces a consistent large reduction (>65%) in Lp(a) concentration with a subsequent improvement in CVD outcomes (e.g., 86% reduction in MACE) [63,64], other lipid-lowering therapeutics have produced heterogeneous effects on Lp(a) and their cardiovascular benefits are mostly absent or remain to be proven. The effect of lipid-lowering therapeutics on Lp(a) range from no response to opposing directions of change (i.e., increases vs. lowering). As noted earlier, statins have generated a highly variable response in Lp(a) in clinical trials [62]. Randomized placebo-controlled clinical trials of anacetrapib, a cholesterol ester transfer protein (CETP) inhibitor, reported a 37% reduction in Lp(a) concentration, but no significant cardiovascular benefit in statin-treated high-risk patients [65]. Another CETP-inhibitor (TA-8995) dose dependently reduced Lp(a) (range: ~27% to 37%) in patients with mild dyslipidemia [66], but its effect on CVD risk is yet to be established. The AIM-HIGH (Atherothrombosis Intervention in Metabolic Syndrome with Low HDL/High Triglyceride and Impact on Global Health Outcomes) trial using a combination of extended-release niacin and statin showed a modest decrease (19%) in Lp(a) compared with the placebo without significant reductions in cardiovascular events [67].

A newer class of lipid-lowering drugs called proprotein convertase subtilisin/kexin 9 (PCSK9) inhibitors has been shown to reduce Lp(a) by ~25% [68] and this Lp(a)-lowering effect was evident across apo(a) size distributions [69]. A post hoc analysis of the FOURIER (Further Cardiovascular Outcomes Research with PCSK9 Inhibition in Subjects with Elevated Risk) trial demonstrated that evolocumab, a PCSK9 inhibitor, reduced Lp(a) in patients with established CVD by ~27% [70]. As expected, elevated Lp(a) concentrations were associated with an increased risk of cardiovascular events irrespective of LDL-C. Notably, patients with higher baseline Lp(a) concentrations experienced greater absolute reductions in their Lp(a) and tended to derive greater coronary benefit (CHD deaths, MI, or urgent revascularization) compared in those with lower baseline concentrations [70]. Evidence from a recent meta-analysis of two PCSK9 inhibitor trials—the FOURIER and ODYSSEY OUTCOMES (Evaluation of Cardiovascular Outcomes after an Acute Coronary Syndrome during Treatment with Alirocumab)—supports Lp(a) as a risk mediator of venous thromboembolism (VTE) as PCSK9 inhibition significantly reduced VTE, which was associated with the degree of Lp(a) lowering, but not LDL-C lowering [71]. The ODYSSEY OUTCOMES trial also reports a similar role for Lp(a) in PAD risk as PCSK9 inhibition with alirocumab reduced the risk of major PAD events by 31%, which was associated with baseline Lp(a), but not LDL-C levels [72]. Furthermore, in the ORION 1 trial (Trial to Evaluate the Effect of ALN-PCSSC Treatment on Low Density Lipoprotein Cholesterol), another PCSK9-modulating agent—inclisiran (a small interference RNA)—resulted in a large interindividual variability in Lp(a) response (−14% to −18% in the single-dose groups and −15% to −26% in the 2-dose groups), which contributed to a non-significant effect of the agent on Lp(a) [73].

Other emerging therapeutics such as those based on antisense oligonucleotide (ASO) targeting apoB-100 or apo(a) appear promising. Addition of mipomersen, an ASO to apoB-100, to a maximal medical therapy in patients with FH reduced Lp(a) by ~26% [74]. An ASO-based approach targeting apo(a) synthesis in the liver reduced Lp(a) concentration by ~35% to 80%, depending on dose and injection frequency, in individuals with established CVD and Lp(a) levels of at least 60 mg/dL [75].

These large reductions in Lp(a) may be the key to testing the Lp(a) hypothesis; the required degree of Lp(a) lowering to meaningfully reduce CHD outcomes has been a subject of debate. A 2018 Mendelian randomization analysis suggested that the clinical benefit of reducing Lp(a) may be proportional to the absolute reduction in Lp(a) concentration and a reduction in Lp(a) of 101.5 mg/dL may be required to produce a clinically relevant reduction in the risk of CHD similar in magnitude to what can be achieved by lowering LDL-C level by 38.67 mg/dL (i.e., 1 mmol/L) [76]. A subsequent 2019 Mendelian randomization analysis estimated that a much lower reduction in Lp(a) (65.7 mg/dL) would be equivalent to a 38.67 mg/dL reduction in LDL-C [77]. The authors noted that the influence of SNPs on Lp(a) concentration and standardization of the Lp(a) assay used may have led to an overestimation (101.5 mg/dL) in the past [77]. More recently, a population-based study concluded that high concentrations of Lp(a) are associated with high risk of recurrent CVD in individuals from the general population and to achieve 20% and 40% MACE risk reduction in secondary prevention, Lp(a) should be lowered by 50 mg/dL and 99 mg/dL for 5 years, respectively [78].

As described, there has been significant investigation of pharmacological intervention for lowering Lp(a) and reducing residual risk conferred by high Lp(a). Heterogeneity is observed in the effect of current lipid-lowering drugs on Lp(a) and the clinical significance is still being investigated. Of note, first-line management of dyslipidemia is a healthy lifestyle including a healthy diet [8]. However, the effect of dietary modification on Lp(a) remains unclear. There is a prevailing perception that dietary modification has no significant effect on Lp(a) (2), which has likely hampered research efforts in this area. There have been several human clinical trials conducted, however that have measured Lp(a) in response to dietary interventions.

## 6. The Effect of Dietary Intervention on Lp(a)

The first report of dietary modulation of Lp(a) was in 1991. In a letter to the editor of *Atherosclerosis*, Hornstra et al. described that in a 2-period, crossover study they observed a 10% reduction in Lp(a) with a palm oil enriched diet (70% replacement of habitual dietary fat with palm oil) compared to a control average Dutch diet after 6 weeks in 38 normolipidemic males [79]. In the three decades since this report, there has been some progress towards understanding dietary regulation of Lp(a) and the underlying mechanisms. In the subsequent sections we will provide an overview of the human clinical trials that have measured changes in Lp(a) in response to diets that are well-defined in terms of the macronutrient composition.

### 6.1. Saturated Fat Replacement

A number of human clinical trials have examined the short-term effect (3–8 weeks) of isocaloric replacement of saturated fat (SFA) with carbohydrate or unsaturated fats on Lp(a) (Table 1). The DELTA (Dietary Effects on Lipoproteins and Thrombogenic Activity) trials comprise the largest examination of the effect of SFA replacement, to date, on Lp(a) [80,81]. DELTA 1 was a randomized, 3-period crossover, controlled feeding study designed to determine the effect of replacing SFA with complex carbohydrate in a normolipidemic cohort [80]. After 8-weeks, lowering of SFA from 15% to 6.1% of calories, with a proportionate increase in complex carbohydrate, increased Lp(a) by ~15% in a dose-response manner. This effect was replicated in the similarly designed DELTA 2 study that randomized individuals who were at risk of CVD. In this trial, replacement of SFA with complex carbohydrate or monounsaturated fat (MUFA) increased Lp(a) by 20% and 11%, respectively [81]. Notably, in both of these trials, LDL-C was reduced by 7–11% with SFA replacement. The findings of the DELTA program provide evidence that lowering SFA intake reduces LDL-C, however, concurrently Lp(a) is increased [80,81].

Increases in Lp(a) have also been observed in other trials where SFA was replaced with MUFA or a combination of MUFA and PUFA [82,83,84,85]. In a 6-week, randomized, crossover, controlled feeding study Clevidence et al. observed an increased in Lp(a) of 9% when ~5% of calories from SFA were replaced with oleic acid in overweight individuals [82]. Interestingly, when subjects were stratified by baseline Lp(a), those with low (≤5 mg/dL), moderate (>5 mg/dL to <30 mg/dL) and high (≥30 mg/dL) levels all had significant increases in Lp(a) of 11.5% (0.3 mg/dL), 20.1% (2.6 mg/dL), and 5.5% (2.8 mg/dL), respectively when SFA was replaced with oleic acid. Clevidence et al. also reported an 8% reduction in LDL-C with replacement of SFA with oleic acid [82]. This is consistent with the findings of a 3-week randomized, crossover, controlled feeding study conducted by Mensink et al. in a cohort of healthy weight adults with normal cholesterol levels [85]. Compared to a diet containing 19% of calories from SFA, a diet higher in oleic acid with 9.5% of calories from SFA increased Lp(a) by 23%, despite lowering LDL-C by 17%. However, in another experiment Mensink et al. did not observe differences in Lp(a) when stearic acid (SFA) was replaced with linoleic acid; LDL-C was reduced by 6% [85]. Further, in a randomized parallel analysis (*n* = 58) where they compared a control high SFA diet to diets lower in SFA and proportionately higher in MUFA or PUFA no significant change in Lp(a) was observed, although the point estimates were increased and it is likely that the analysis was underpowered to detect this effect [85].

In two more recent analyses, reductions in Lp(a) were observed when SFA was replaced with carbohydrate or unsaturated fat [86,87]. A 3-period, randomized crossover controlled feeding study showed that a low-fat diet (CHO: 59% kcal; fat: 24% kcal; SFA: 7% kcal) and a moderate fat diet (CHO: 49% kcal; fat: 34% kcal; SFA: 6% kcal) lowered Lp(a) relative to baseline where subjects were consuming an average American run-in diet (CHO: 51%; 34% kcal fat; 13% kcal SFA) [87]. However, a moderate fat diet containing avocado did not reduce Lp(a) from baseline; Lp(a) was 6% lower with the low-fat diet relative to the avocado containing higher fat diet. Similarly, in a 3-period, randomized crossover, controlled feeding study, Tindall et al. observed a 11.5% reduction in Lp(a) with a diet higher PUFA relative to baseline where subjects were consuming a high SFA average American diet; no change in Lp(a) was detected with a macronutrient matched diet containing walnuts or a diet higher in MUFA [86]. It is unclear why Lp(a) lowering was observed in these two controlled feeding studies when earlier studies showed increases in Lp(a) with similar SFA replacement [80,81,82,83,84,85]. Of note, in both studies, Lp(a) was measured by the vertical auto profile (VAP) method that uses ultracentrifugation to quantify lipoprotein concentration based on flotation rate [88]. This method measures cholesterol concentration of Lp(a) particles instead of apo(a) or Lp(a) particle concentration [88]. There is a poor correlation of VAP measured values with Lp(a) mass, raising concerns regarding the use of this method due to potential overlap with other lipoprotein fractions, including HDL [89].

### 6.2. Diets with Different Macronutrient Compositions

The effect of diets with differing macronutrient compositions on Lp(a) has also been examined in several human clinical trials (Table 2). Omni Heart (Optimal Macronutrient Intake Trial to Prevent Heart Disease) was a 3-period, randomized, crossover, controlled feeding study that examined the effect of three Dietary Approaches to Stop Hypertension (DASH)-style diets with differing macronutrient compositions, a high carbohydrate diet, a high-protein diet and a diet high in unsaturated fat [90]. In this cohort with elevated blood pressure, Lp(a) was increased from baseline with all three diets (~8–18%). Notably, the higher protein diet increased Lp(a) more than the higher carbohydrate diet (1.4 mg/dL) and the higher unsaturated fat diet (2.5 mg/dL). Furthermore, the unsaturated fat diet increased Lp(a) less than the higher carbohydrate (−1.1 mg/dL) diet. In this study, just over half the cohort were Black, and a larger increase in Lp(a) was observed in Blacks vs. Whites following the higher protein diet (6.2 vs. 2.6 mg/dL); there was no difference in the Lp(a) response by race for the diet higher in unsaturated fat or the higher carbohydrate diet. This study showed LDL-C lowering with all three diets [91].

A number of studies have examined the effect of low-fat, higher carbohydrate diets compared to high-fat, lower carbohydrate diets on Lp(a). Faghihnia et al. reported that relative to a high-fat diet (fat 40% kcal; SFA 13%), a low-fat diet (fat 20%; SFA 5%) increased Lp(a) by ~12% after 4 weeks [92]. Increases in OxPL per apoB (451 relative light units (RLU)) and apo(a) (178 RLU), and triglycerides (31 mg/dL) and apoB (5.2 mg/dL) were also observed with the low-fat diet compared with the high-fat diet. However, LDL-C (−6.6 mg/dL), apoA-1 (−5.0 mg/dL) and HDL-C (−4.1 mg/dL) were lower. The reduction in LDL-C with the low-fat diet appeared to be because of a shift in the LDL particle distribution; the low-fat diet reduced the concentration of larger LDL particles and increased smaller LDL particles and, therefore, overall LDL peak particle diameter was reduced. Interestingly, the change in Lp(a) was positively associated with the change in OxPL per apoB and medium sized LDL (LDL II), and inversely related to the change in small LDL (LDL IV) [92]. This suggests that diet-induced changes in Lp(a) and LDL particles may be related; however, the evidence in support of this is limited with some discrepancies. In the previously described study conducted by Wang et al., a low-fat diet reduced Lp(a) relative to baseline, and a reduction in LDL II and an increase in LDL IV was observed [87]. The shift in LDL II and IV with the low-fat diet is consistent with the findings reported by Faghihnia et al., although the Lp(a) finding is directionally opposite. In addition, Tindall et al.’s findings also contrast with the Lp(a) and LDL particle findings previously described. Following a higher PUFA diet, Tindall et al. observed a reduction in Lp(a) and a reduction in the concentration of large LDL (I and II) compared to baseline; no changes were detected in smaller LDL (III and IV) [86]. Finally, Berryman et al. reported that Lp(a) was lower (1 mg/dL) following a lower fat, higher carbohydrate diet compared to a higher fat diet containing 43 g/day of almonds [93]. However, LDL-C was lower following the high fat diet with almonds relative to the lower fat diet (5 mg/dL); no between-diet differences were detected in the concentrations of LDL particles. Thus, inconsistencies exist in the findings of the few diet studies measuring changes in Lp(a) and LDL particle concentrations, which is partly attributable to differences in analytical approaches. Therefore, further investigation of the relationship between diet-induced changes in LDL particles and Lp(a) is required to understand these contrasting results and the atherogenicity conferred by such changes.

A few studies have investigated the effects of diets enriched with nuts with varying macronutrient compositions. Jenkins et al. found that a higher fat diet with 73 g/day of almonds reduced Lp(a) relative to a control diet lower in fat but matched for SFA; no difference in Lp(a) was observed with 37 g/day of almonds [94]. Rajaram et al., observed a 15% reduction in Lp(a) with a pecan-enriched diet (20% kcal; 72 g/day/2400 kcal) compared to a higher carbohydrate, lower fat control diet; LDL-C was reduced by 10% with the pecan diet vs. the control [95]. Similarly, in a 2 period, randomized, crossover trial a Mediterranean diet with walnuts (41–56 g/day) reduced Lp(a) and LDL-C by 6% compared to a control Mediterranean diet without walnuts after 6 weeks [96]. Finally, Lee et al. reported no change in Lp(a) with an almond enriched diet (42.5 g/day), a chocolate enriched diet (61 g/day) or an almond + chocolate enriched diet compared to a diet representative of average American macronutrient intake [97]. Together these studies suggest that Lp(a) modulation may go beyond the macronutrient profile of a diet and be affected by foods and other non-macronutrient dietary components. Further investigation of the effect of complete well-characterized dietary patterns on Lp(a) is required.

In summary, most of the available evidence suggests that replacement of SFA with carbohydrate or unsaturated fat modestly increases Lp(a), while consistently decreasing LDL-C. Although the results of a few trials deviate from this and suggest replacement of SFA with unsaturated fat from particular food sources such as nuts may not increase Lp(a). However, the few trials identified and the measurement of Lp(a) as a secondary endpoint in the majority of studies highlights that further investigation of dietary modulation of Lp(a) is needed. This review has focused on evidence from human clinical trials examining the effect of well-defined diets on Lp(a). Other studies have investigated the effect of dietary supplements (L-carnitine, and coenzyme Q10) and specific foods (coffee, tea and alcoholic beverages, especially red wine) and have shown decreases in Lp(a) with these interventions; this research, however is beyond the scope of this review and was summarized recently by Santos et al. [98]. Collectively, the studies reviewed herein and the additional studies that have been conducted with supplements and certain foods demonstrate a modulating effect of diet on Lp(a). 

## 7. Potential Mechanisms to Explain Pharmacological and Non-Pharmacological (e.g., Diet) Intervention-Induced Changes in Lp(a) Concentration

**Figure 2:**
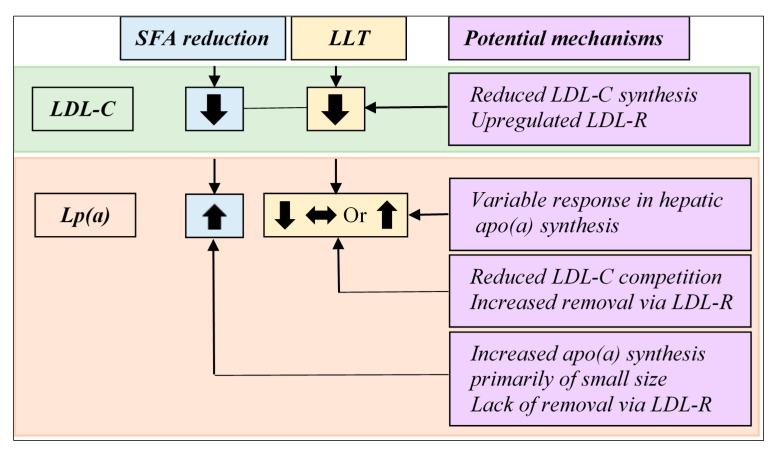
Effect on LDL-C vs. Lp(a) by lowering of dietary saturated fat intake vs. lipid-lowering therapy and potential underlying mechanisms. While both lipid-lowering therapy (LLT) and reduction in dietary saturated fatty acid (SFA) intake lower plasma LDL-C concentrations, their effects on Lp(a) vary. Lowering dietary SFA intake has been associated with a modest increase in Lp(a) concentration. The effect of existing LLT on Lp(a) concentration is heterogeneous. Statins induce either an increase or a reduction, whereas inhibitors of CETP or PCSK9 have been associated with decreases in Lp(a). Lp(a) plasma concentration is primarily regulated by apo(a) synthesis in the liver and the role of LDL receptor (LDL-R) in Lp(a) metabolism remains incompletely understood.

The plasma concentration of Lp(a) is primarily determined by the synthesis of apo(a) in the liver, where the production rate is genetically controlled through a copy number variation (i.e., the apo(a) size polymorphism). The role of LDL receptor (LDL-R)-mediated catabolism in Lp(a) homeostasis remains debatable. Statins that exert their effects through upregulating LDL-R have shown mixed effects on Lp(a) [62] with some trials even reporting selective increases in Lp(a) in carriers of a small size apo(a) [99]. An overall increased awareness of a heart healthy lifestyle and reducing SFA intake among patients initiating statin therapy may contribute to these findings [100]. Findings in clinical trials with PCSK9 inhibitors add complexity to this matter as PCSK9 inhibitors have been associated with a modest but consistent reduction in Lp(a) [68,69] despite their function to promote LDL-R recycling, thereby, increasing the number of available LDL-R on the cell surface [101]. These observations suggest a possible role for LDL-R-mediated catabolism in Lp(a) reduction during certain pharmacological interventions. It is tempting to speculate that under physiological conditions hepatic apo(a) synthesis remains as the key regulator of Lp(a) homeostasis, whereas under pharmacological interventions, where, for example, LDL-C is reduced to a very low level, Lp(a) can be cleared by LDL-R, allowing manipulation through catabolism. Observed increases in Lp(a) during dietary SFA reduction, a common non-pharmacological intervention [81,90], likely is a result of increased hepatic apo(a) synthesis rather than a reduced catabolism of Lp(a). In support of this, in a randomized crossover study of cynomolgus monkeys, Lp(a) was lowered when SFA was replaced with MUFA and there was a concomitant reduction in hepatic apo(a) mRNA abundance, suggesting a reduction in apo(a) transcription [102]. The finding of lowered Lp(a) following replacement of SFA with MUFA in this early study of cynomolgus monkeys, which are known to have Lp(a) with similar immunologic properties to humans [103], is directionally opposite to most of the more recent human trials, although suggests dietary fatty acids regulate hepatic apo(a) synthesis affecting Lp(a) levels. The authors are not aware of any other animal studies examining dietary regulation of Lp(a). Of note, Lp(a) has only been detected in humans, nonhuman primates, and hedgehogs [104,105]. Figure 2 compares the effects of dietary SFA reduction vs. lipid-lowering therapeutics on LDL-C vs. Lp(a) and proposes potential mechanisms underlying their differential effects. Future mechanistic as well as clinical studies of emerging therapeutics may provide new insights into the roles of Lp(a)/apo(a) synthesis, catabolism, or both in Lp(a) regulation and manipulation.

## 8. Measurement of Low-Density Lipoprotein Cholesterol (LDL-C) and Lp(a) Change

**Figure 3:**
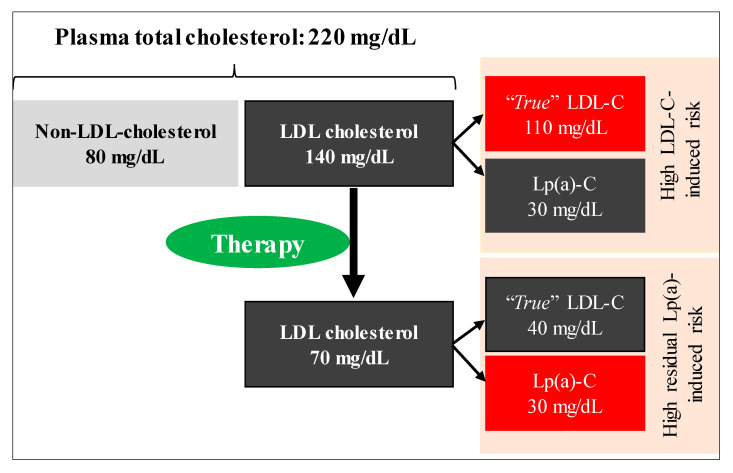
A hypothetical case describing the Lp(a)-associated residual cardiovascular risk following a therapy. Clinical measurement of LDL-C includes cholesterol carried on Lp(a) (~30% of Lp(a) mass). An individual with an LDL-C level of 140 mg/dL, which includes 30 mg/dL cholesterol carried on Lp(a), reduced LDL-C to 70 mg/dL with a therapy. While LDL-C-attributable CVD risk is controlled, Lp(a)-associated residual risk remains high. Lowering dietary saturated fat intake, which is a recommended therapy, increases Lp(a) concentration, thus may promote Lp(a)-induced residual risk even further.

Compositional analysis indicates that Lp(a) is composed of ~30% (or more) cholesterol [106] and this amount is included in current clinical measurements of LDL-C. This will likely result in an under- or over- estimation of the “*true*” LDL-C response to pharmacological and non-pharmacological interventions where changes occur in both LDL-C and Lp(a). Examples include statin trials that increase (or reduce) Lp(a) [62] and metabolic feeding trials where SFA is replaced with other macronutrients and commonly results in an increase in Lp(a) [81,90], despite clinically relevant reductions in LDL-C. To obtain a more accurate estimation of the effect on LDL-C, independent of Lp(a) change, there is a need to correct LDL-C values for the contribution of Lp(a) cholesterol. We and others have calculated the *true* LDL-C (i.e., corrected LDL-C) value by multiplying Lp(a) mass (mg/dL) by 0.30 to derive Lp(a) cholesterol, then subtracting this value from the measured LDL-C value [62,107]. This issue could become even more relevant in situations where interventions are tested in a diverse group of individuals, e.g., African-Americans, patients with FH, who generally have higher Lp(a) levels. Figure 3 shows a hypothetical case describing the Lp(a)-induced residual cardiovascular risk in relation to *true* LDL-C concept following a therapy.

## 9. Future Direction

In this review, we have provided an up-to-date summary of the CVD risk-enhancing role of Lp(a) and the effect of established lipid-lowering therapies to lower Lp(a). In addition, this paper presents the first in-depth examination of human clinical trial evidence on the effect of dietary interventions on Lp(a). In the ~30 years since an effect of diet on Lp(a) was first reported, only a relatively small number of studies have examined changes in Lp(a) in response to diet. We have summarized 14 studies that reported the macronutrient profile of the test diets since, presently, macronutrient substitution is thought to affect Lp(a), particularly SFA replacement. For comparison, dietary macronutrient profile is a well-established predictor of total cholesterol and LDL-C change, and the most recent synthesis of evidence from controlled feeding studies of SFA replacement included 84 studies [5]. Thus, to characterize the effect of dietary interventions on Lp(a) greater evidence is needed. Of particular importance is that dietary interventions are well-defined and reported with regard to the macronutrient profile and the foods included in the study menus. In addition, the diet studies identified in the review used a variety of methods to measure Lp(a) and no studies reported the results as a particle concentration. It is recommended that Lp(a) be measured as a particle concentration because, unlike other lipids and lipoproteins, the isoforms have different molecular weights [59]. Furthermore, given the challenges of comparing outcomes using different analytical approaches, particularly relevant for Lp(a), it is recommended that an immunochemical assay calibrated against the World Health Organization International Federation of Clinical Chemistry and Laboratory Medicine secondary reference material [108] is used for Lp(a) measurement [59].

As described, often in response to dietary change and some pharmacological agents Lp(a) is increased in the context of LDL-C lowering. Presently, the clinical significance of the discordance in Lp(a) and LDL-C responsiveness is not well-understood. Further investigation into the CVD risk associated with increased Lp(a) in the presence of LDL-C lowering is needed. In particular, characterization of the atherogenic properties of the Lp(a) particle is needed. In addition, a greater understanding of the heterogeneity in Lp(a) responsiveness to diet or pharmacological therapies by apo(a) size, race/ethnicity, metabolic phenotype and LDL-C change is required. In the meantime, there is insufficient evidence to make dietary recommendations for patients with high Lp(a) and, therefore, patients should continue to be advised to replace SFA with unsaturated fat consistent with current recommendations for the prevention and management of dyslipidemia to reduce CVD risk [6,7,109,110].

## 10. Conclusions

There is a renewed interest in Lp(a) as a clinical indicator of CVD risk and a potential treatment target. While new pharmacological therapeutics show promise in lowering Lp(a), the clinical significance is still being evaluated. In terms of non-pharmacological therapy, there is a well-established dogma that diet has no effect on Lp(a) and to date there have been few well-controlled clinical investigations of the effect of dietary modification on Lp(a). We have summarized the evidence to date, which suggests that dietary interventions affect Lp(a), although often Lp(a) is increased especially when SFA is replaced by other macronutrients; the clinical significance of this increase is unclear. In addition, we identified heterogeneity in the reported dietary interventions, methods used to measure Lp(a), and a lack of research about the underlying mechanisms. Therefore, further investigation of the effect of well-defined diets is needed to examine dietary modulation of Lp(a). Finally, it will be important to evaluate whether diet-induced Lp(a) effects are modified by other biological (e.g., race/ethnicity), genetic (e.g., apo(a) size) and metabolic (high vs. low burden) phenotypes. These findings will help prevention and treatment guidelines to evolve in order to further reduce CVD risk.

## Figures and Tables

**Table 1:** The effect of replacing saturated fat with other macronutrients on Lp(a) and low-density lipoprotein cholesterol (LDL-C).

Study	Design	Study Duration	Participants	*N*	Test Diets	Macronutrient Profiles of the Test Diets ^1^						Lp(a) mg/dL (Mean ± SEM)	LDL-C (Measured) mg/Dl (Mean ± SEM)	SFA Replacement Effect Summary
						CHO	PRO	Total Fat	SFA	MUFA	PUFA			
Ginsberg et al. 1998 (USA) DELTA 1 [80]	4-site multicenter, randomized, 3 period crossover, controlled feeding trial	8-week diet periods (4–6 washout)	Normolipidemic, aged 22–65 years; 55% women (30% black; 32% postmenopausal) and 45% men (20% black)	103	AAD	48	15	34.3	15.0	12.8	6.5	15.5 ± 1.8 ^a^	131.4 ± 2.7 ^2,a^	SFA→CHO: ↑ Lp(a) ↓LDL-C
					Step 1	55	15	28.6	9	12.9	6.7	17.0 ± 1.8 ^b^	122.2 ± 2.6 ^2,b^	
					Low-SFA	59	15	25.3	6.1	12.4	6.7	18.2 ± 1.9 ^c^	116.9 ± 2.6 ^2,c^	
Berglund et al. 2007 (USA) DELTA 2 [81]	4-site multicenter, randomized, 3 period crossover, controlled feeding trial	7-week diet periods (4–6 washout)	Low HDL-C, moderately elevated triglycerides and insulin;aged 21–65 years; 39% women (18% black) and 61% men (8% black)	85	AAD	49	15.3	35.8	15.6	14.4	5.8	9.9 ± 1.4 ^a^	128 ± 3.1 ^a^	SFA→CHO: ↑ Lp(a) ↓LDL-C SFA→MUFA: ↑ Lp(a) ↓LDL-C
					MUFA	48.8	15.5	35.7	8.7	20.8	6.2	11.0 ± 1.5 ^b^	120 ± 3.1 ^b^	
					Step 1	54.9	16.1	29	8	15.5	5.5	11.9 ± 1.6 ^b^	119 ± 3.1 ^b^	
Clevidence et al. 1997 (USA) [82]	Randomized, 4 period, crossover, controlled feeding trial	6-week diet periods (no washout)	80–120% of desirable BMI; aged 25–65 years; total cholesterol 50th–75th percentile; HDL-C > 35 mg/dl (men) or >40 mg/dL (women)	58	SFA	45	15	40	19.3 ^4^	10.9 ^5^	6.1 ^6^	21.9 ± 0.4 ^a^	141 ± 9.3 ^2,a^	SFA→MUFA: ↑ Lp(a) ↓LDL-C
					Oleic	46	15	39	13.4 ^4^	16.7 ^5^	6.1 ^6^	23.8 ± 0.4 ^b^	129 ± 9.3 ^2,b^	
					Moderate trans fat ^7^	46	15	39	13.0 ^4^	14.1 ^5^	6.0 ^6^	23.8 ± 0.4 ^b^	137 ± 9.3 ^2,c^	
					High trans fat ^8^	46	15	39	12.7 ^4^	11.4 ^5^	6.2 ^6^	24.7 ± 0.4 ^b^	139 ± 9.3 ^2,a,c^	
Mensink et al. 1992 (The Netherlands) Experiment 1 [85]	2 group parallel, controlled feeding trial	17 days–control run-in diet 36 days–MUFA or PUFA	Young, normolipidemic (mean total cholesterol 193 ± 31 mg/dL), non-obese (mean BMI 21.6 ± 2.0 kg/m^2^) students	58	High SFA (control)	48–49	13	36.7	19.3	11.5	4.6	Pre MUFA: 8.4 (0–34.0) ^9^ Pre PUFA: 3.7 (0–23.5) ^9^	Pre MUFA: 128 ± 29 ^2,3^ Pre PUFA: 129 ± 26 ^2,3^	SFA→MUFA: ←→ Lp(a) ↓LDL-C SFA→PUFA: ←→ Lp(a) ↓LDL-C
				29	MUFA	48–49	13	37.4	12.9	15.1	7.9	9.1 (0–33.6) ^9^	104 ± 26 ^2,3,a^	
				29	PUFA	48–49	13	37.6	12.6	10.8	12.7	4.0 (0–24.0) ^9^	111 ± 23 ^2,3,b^	
Mensink et al. 1992 (The Netherlands) Experiment 2 [85]	Randomized, 3 period, crossover, controlled feeding trial	3-week diet periods (washout not reported)	Mean total cholesterol 184 ± 31 mg/dL; Mean BMI 21.5 ± 2.1 kg/m^2^	59	SFA	46	13–14	38.8	19.4	14.7	3.4	2.6 (0–44.7) ^9,a^	121 ± 22 ^2,3,a^	SFA→MUFA: ↑ Lp(a) ↓LDL-C
					Oleic acid ^10^	46	13–14	39.6	9.5	24.1	4.6	3.2 (0–48.4) ^9,b^	103 ± 21 ^2,3,b^	
					Trans fat	46	13–14	40.2	10.0	13.3	4.6	4.5 (0–51.0) ^9,c^	118 ± 24 ^2,3c^	
Mensink et al. 1992 (The Netherlands) Experiment 3 [85]	Randomized, 3 period, crossover, controlled feeding trial	3-week diet periods (washout not reported)	Mean total cholesterol 195 ± 25 mg/dL; Mean BMI 22.0 ± 2.3 kg/m^2^	56	Stearate	44–47	12–13	43.5	20.1 (11.8 stearic acid)	16.3	4.3	6.9 (0–74.9) ^9,a^	116 ± 27 ^2,3,a^	SFA→PUFA: ←→ Lp(a) ↓LDL-C
					Linoleate	44–47	12–13	41.1	11.0 (2.8 stearic acid)	15.7	12.5	6.9 (0–78.2) ^9,a^	109 ± 24 ^2,3,b^	
					Trans fat ^11^	44–47	12–13	39.7	10.3 (3.0 stearic acid)	15.6	3.8	8.5 (0–89.1) ^9,b^	119 ± 25 ^2,3,a^	
Muller et al. 2003 (Norway) [83,84]	Randomized, 3 period, crossover, controlled feeding trial	3-week diet periods (1-week washout)	Female students, aged 31 ± 10, BMI 24.5 ± 3.2 kg/m^2^	25	High saturated fat	46.7	14.9	38.4	22.7 ^12^	5.5	3.9	31.6 ± 48.7 ^3,a^	124 ± 30 ^2,3,a^	SFA ^12^→MUFA/PUFA: ↑ Lp(a) ↓LDL-C SFA ^12^→CHO: ←→ Lp(a) ←→ LDL-C
					Low saturated fat	63.8	16.5	19.7	10.5 ^12^	3.5	2.3	34.0 ± 49.3 ^3,a,b^	121 ± 26 ^2,3,a^	
					High MUFA/PUFA	46.8	15	38.2	2.4 ^12^	14.1	15.6	35.8 ± 51.5 ^3,b^	97 ± 25 ^2,3,b^	

1. Percentage of total kcal, unless otherwise stated, 2. Calculated LDL-C (Friedewald equation) or method not reported, 3. Standard deviation, 4. Lauric + myristic + palmitic + stearic acids only, 5. Oleic acid only, 6. Linoleic acid only, 7. Contains 3.8% kcal from trans fat, 8. Contains 6.6% kcal from trans fat, 9. Median (range), 10. Trans oleic acid 10.9% kcal, 11. Trans oleic acid 7.7% kcal, 12. Only C12:0, C14:0, C16:0 (from coconut oil), *Different vs. baseline (or run-in), LDL_real_ is LDL-C minus Lp(a) and IDL, For a study, values with differing superscript letters for an outcome are statistically different (*p* < 0.05). Abbreviations: AAD Average American Diet; BMI body mass index; CHO carbohydrate; HDL-C high density lipoprotein cholesterol; Lp(a) lipoprotein(a); LDL-C low-density lipoprotein cholesterol; MUFA monounsaturated fatty acids; PRO protein; PUFA polyunsaturated fatty acids; SFA saturated fatty acids

**Table 2:** The effect of diets with differing macronutrient compositions of Lp(a) and LDL-C.

Study	Design	Study Duration	Participants	*N*	Test diets	Macronutrient Profiles of the Test Diets ^1^						Lp(a) mg/dL (Mean ± SEM)	LDL-C (Measured) mg/dL (Mean ± SEM)	Effect Summary
						CHO	PRO	Total Fat	SFA	MUFA	PUFA			
Omni Heart (USA) [90,91]	Randomized, 3 period, crossover, controlled feeding trial	6-week diet periods (2–4 week washout)	Systolic blood pressure 120–159 mmHg or diastolic blood pressure 80–99 mmHg, aged > 30 years; 46% women (70% black) and 54% men (44% black)	155	CHO	58	15	27	6	13	8	3.2 (2.2, 4.2) ^2,^*^,a^	-11.6 (-14.6, -8.6) ^2,3,^*^,a^	CHO→PRO ↑ Lp(a) ↓LDL-C CHO→MUFA/PUFA ↑ Lp(a) ↓LDL-C MUFA/PUFA→PRO ↑ Lp(a) ↓LDL-C
					Protein	48	25	27	6	13	8	4.7 (3.7, 5.7) ^2,^ *^,b^	-14.2 (-17.5 -10.9) ^2,3,^*^,b^	
					Unsaturated fat	48	15	37	6	21	10	2.1 (1.1, 3.1) ^2,^*^,c^	-13.1 (-16.4, -9.8) ^2,3,^*^,a,b^	
Faghihnia et al. 2010 (USA) [92]	Randomized, 2 period, crossover, trial	4-week diet periods (no washout)	Body weight <130% of ideal; aged >20 years; 97% men	63	High-fat, low-CHO	45	15	40	13	11	14	17.8 ± 12.8 ^4,a^	124.0 ± 31.5 ^3,4,a^	High-fat, low-CHO → Low-fat, high-CHO ↑ Lp(a) ↓LDL-C
					Low-fat, high-CHO	65	15	20	5	10	5	19.9 ± 13.7 ^4,b^	117.3 ± 30.7 ^3,4,b^	
Berryman et al. 2015 (USA)) [93]	Randomized, 2 period crossover, controlled feeding trial	6-week diet periods (2-week washout)	LDL-C 121–190 mg/dL women or 128–194 mg/dL men; aged 30–65 years; BMI 20–35 kg/m^2^; 54% women	48	Almond	51.3	16.4	32.3	7.7	13.9	8.4	7.7 ± 0.8 ^a^	129 ± 3 ^a^	Lower fat, higher CHO → Higher fat, lower CHO diet with almonds ↑ Lp(a) ↓LDL-C
					Control	58.4	15.2	26.4	7.8	10.4	6.2	6.7 ± 0.8 ^b^	135 ± 3 ^b^	
Jenkins et al. 2002 (Canada) [94]	Randomized, 3 period crossover, trial	4-week diet periods (>2-week washout)	Hyperlipidemic (LDL-C >159 mg/dL); aged 48–86 years; BMI 20.5–31.5 kg/m^2^; 56% men and 44% postmenopausal women	27	Full-dose almond	44.8	17.4	36.0	7.2	18.9	8.2	14.2 ± 2.9 ^a^	155 ± 4.6 ^3,a^	Lower fat, higher CHO → Higher fat, lower CHO diet with almonds ↓ Lp(a) ↓LDL-C
					Half-dose almond	48.4	17.6	32.1	7.5	14.5	8.0	15.4 ± 3.2	159 ± 4.6 ^3,a^	
					Control	54.5	17.5	26.3	7.0	9.0	8.0	15.5 ± 3.2 ^b^	163 ± 5.0 ^3,b^	
Lee et al. 2017 (USA) [97]	Randomized, 4 period crossover, controlled feeding trial	4-week diet periods (2-week washout)	Overweight or obese; aged 30–70 years; LDL-C 25th–95th percentile	31	AAD	49	17	34	13	13	7	4.9 (4.1, 5.8) ^5^	135.6 ± 2.8 ^a^	Average American diet → Higher fat, lower saturated fat diet with almonds or almonds + chocolate ←→ Lp(a) ↓LDL-C Average American diet → higher carbohydrate diet with chocolate ←→ Lp(a) ←→LDL-C
					Almond	48	16	36	8	16	9	5.3 (4.5, 6.3) ^5^	126.4 ± 2.8 ^b^	
					CHOC	51	16	33	12	12	6	4.6 (3.9, 5.5) ^5^	136.1 ± 2.8 ^a^	
					Almond+ CHOC	49	16	35	9	9	8	5.1 (4.3, 6.1) ^5^	128.9 ± 2.8 ^b^	
Rajaram et al. 2001 (USA) [95]	Randomized, 2 period crossover, controlled feeding trial	4-week diet periods (no washout)	Healthy; total cholesterol 15th–80th percentile	23	Step 1	56.8	14.5	28.3	8.2	11.0	6.3	25 ± 22 ^4,a^	117.9 ± 21.7 ^4,6,a^	Pecan-enriched higher fat, lower carbohydrate diet → lower fat, higher carbohydrate diet ↓ Lp(a) ↓LDL-C
					Pecan-enriched	47.2	13.1	39.6	8.1	18.9	10.7	21 ± 18 ^4,b^	105.6 ± 19.7 ^4,6,b^	
Zambon et al. (Spain) [96]	Randomized, 2 period crossover, controlled feeding trial	4-week diet periods (no washout)	Polygenic hypercholesterolemia	49	Control (Mediterranean)	49.8	19.0	31.2	6.9	17.5	4.8	34 ± 24 ^4,a^	185 ± 25 ^4,a^	Mediterranean diet → Mediterranean diet with walnuts (35% of total fat; 41–56 g/day) ↓ Lp(a) ↓LDL-C
					Walnut (Mediterranean)	48	17.9	33.2	6.0	13.5	11.7	32 ± 22 ^4,b^	174 ± 30 ^4,b^	

1. Percentage of total kcal, unless otherwise stated, 2. Change from baseline; mean (95% CI), 3. Calculated LDL-C (Friedewald equation) or method not reported, 4. Mean ± standard deviation, 5. Geometric mean (95% CI), * different vs. baseline (or run-in), For a study, values with differing superscript letters for an outcome are statistically different (*p* < 0.05). Abbreviations: AAD Average American Diet; BMI body mass index; CHO carbohydrate; CHOC enriched chocolate diet; Lp(a) lipoprotein(a); LDL-C low-density lipoprotein cholesterol; MUFA monounsaturated fatty acids; PRO protein; PUFA polyunsaturated fatty acids; SFA saturated fatty acids
